# Transcaval transcatheter aortic valve implantation in bicuspid aortic valve: A step‐by‐step procedural guidance

**DOI:** 10.1111/jocs.16735

**Published:** 2022-07-16

**Authors:** Mi Chen, Jonathan Michel, Barbara E. Stähli, Christian Templin, Philipp Jakob, Felix C. Tanner, Albert Markus Kasel

**Affiliations:** ^1^ Department of Cardiology University Hospital Zurich Zurich Switzerland; ^2^ Department of Cardiac Surgery, Beijing Anzhen Hospital Capital Medical University Beijing China

**Keywords:** bicuspid aortic valve, transcatheter aortic valve implantation (TAVI), transcaval TAVI

## Abstract

We report the case of a 78‐year‐old female with Sapien 3 transcatheter heart valve implantation in the transcaval approach. In this setting, we describe the step‐by‐step management and technique of the transcaval transcatheter aortic valve implantation.

## Key Points

- Anatomical selection, cava‐aorta puncture, and shunt occlusion play key roles in the transcaval approach.
- Predicted projections of cava‐aorta access on long and short axes simplify the perpendicular puncture.

**Abbreviations**
BAV: bicuspid aortic valve
CT: computed tomography
IVC: inferior vena cava
TAVI: transcatheter aortic valve implantation
THV: transcatheter heart valve

## 1 INTRODUCTION

When the transfemoral arterial approach is invalid, the transfemoral approach via vein with transcaval crossing is regarded as an alternative minimally invasive approach for TAVI.^1^ We report the case of a 78‐year‐old female with Sapien 3 transcatheter heart valve (THV) implantation in the transcaval approach. In this setting, we describe the step‐by‐step management and technique of the transcaval transcatheter aortic valve implantation (TAVI). TAVI indication was driven by the institutional heart team and the patient provided written informed consent before the procedure. The patient was included in the nationwide Swiss TAVI Registry (NCT01368250) approved by local ethic committees.

## 2 CASE REPORT

### 2.1 Patient history

A 78‐year‐old female with coronary artery disease, hypertension, and carotid artery stenosis presented to the cardiology department complaining of dyspnea.

### 2.2 Investigations

Transthoracic echocardiography (TTE) showed severe aortic stenosis (mean transaortic pressure gradient = 53 mmHg; aortic valve area = 0.6 cm^2^; indexed aortic valve area = 0.43 cm^2^/m^2^; aortic valve *V*_max_ = 4.6 m/s; left ventricular ejection fraction = 63%). Coronary angiography showed 60% stenosis of left anterior artery.

#### 2.2.1 Pre‐TAVI CT evaluation

Pre‐TAVI computed tomography (CT) demonstrated a type‐1 (L−R) bicuspid aortic valve (BAV) with an aortic annulus area of 441.3 mm^2^ and area‐derived diameter of 23.7 mm. Severe stenosis and former stent implantation of bilateral femoral arteries contraindicated to transfemoral approach.

#### 2.2.2 Anatomic evaluation for transcaval approach

**Figure 1:**
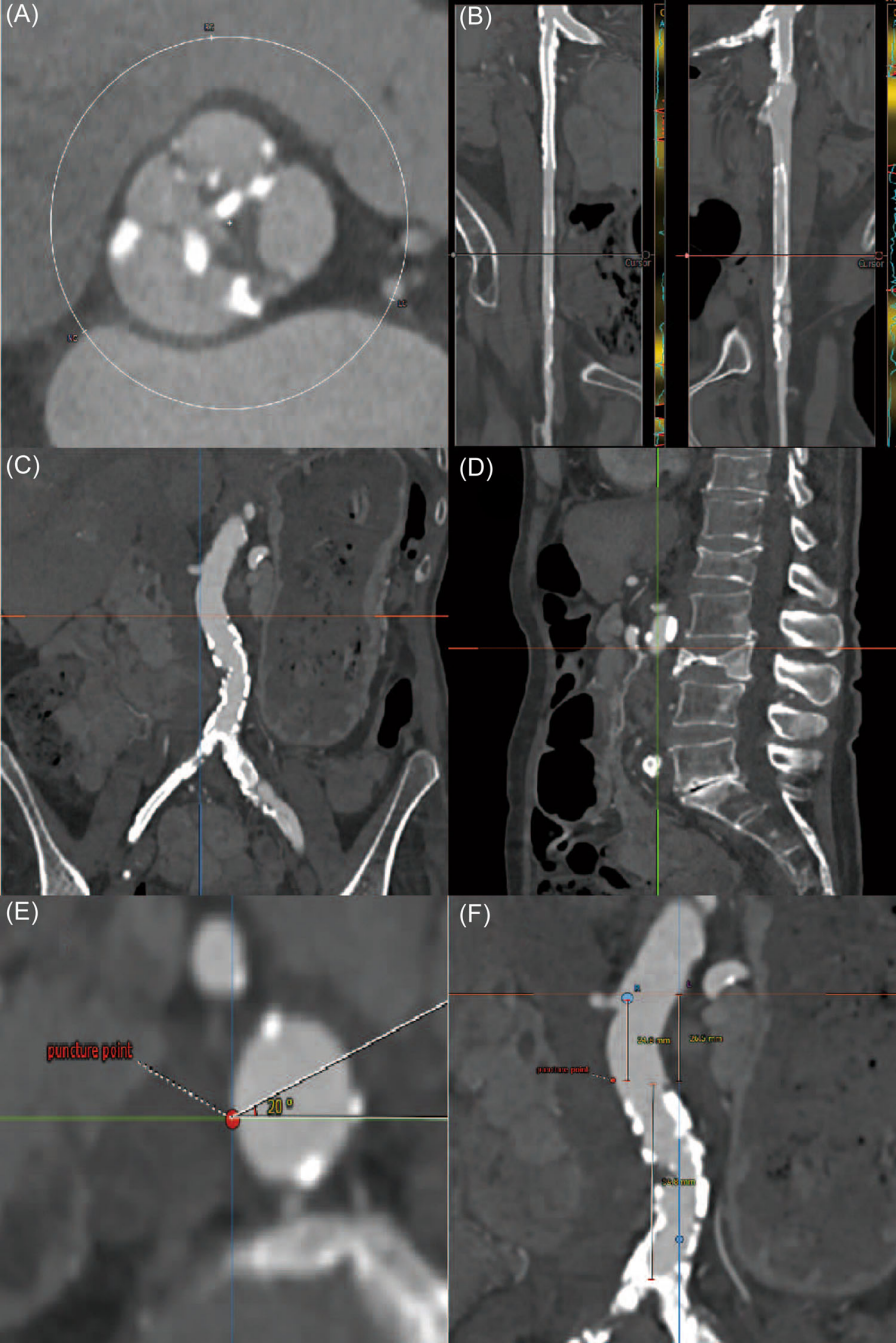
Pre‐TAVI CT evaluation. Pre‐TAVI CT showed Type‐1 bicuspid aortic valve (L−R) (A) and invalid transfemoral access (B). The ideal cava‐aorta puncture site was defined in the nearest location between IVC and aorta without calcification in the aortic wall (C). The upper part of L3 was defined as the bone marker for a puncture (D). The angle of the perpendicular puncture was measured to predict the long axis and short axis projections (E). The distances of the puncture site to renal arteries and iliac bifurcation were measured to ensure enough length for an emergent stent implant (F). CT, computed tomography; IVC, inferior vena cava; TAVI, transcatheter aortic valve implantation

An ideal puncture site of cava‐aorta access is defined as (a) the nearest site of inferior vena cava (IVC) and the abdominal artery; (b) a calcium‐free crossing site; a minimal distance of 10 mm to renal arteries and aortic bifurcation (c) and (d) remarkable bone reference marker. In this case, the upper part of the compressed vertebral body L3 was defined as the ideal puncture site. RAO 20° as the long axis and LAO 70° as the short axis were predicted as puncture projections. Additionally, the distances between the puncture site to renal arteries or bifurcation of iliac arteries were measured to ensure enough length for bailout stent implantation (Figure 1).

### 2.3 Management

The Heart Team decided to proceed with TAVI, given the patient's age, frailty, and the burden of comorbidities. The transcaval approach was selected given the severe peripheral artery disease. Based on the annular anatomy, TAVI with a 26‐mm Sapien 3 (Edwards Lifesciences) balloon‐expandable THV with 23‐mm balloon‐sizing predilatation was planned. This procedure was under general anesthesia with simultaneous transesophageal echocardiography (TEE) evaluation.

#### 2.3.1 Cava‐aorta puncture

**Figure 2:**
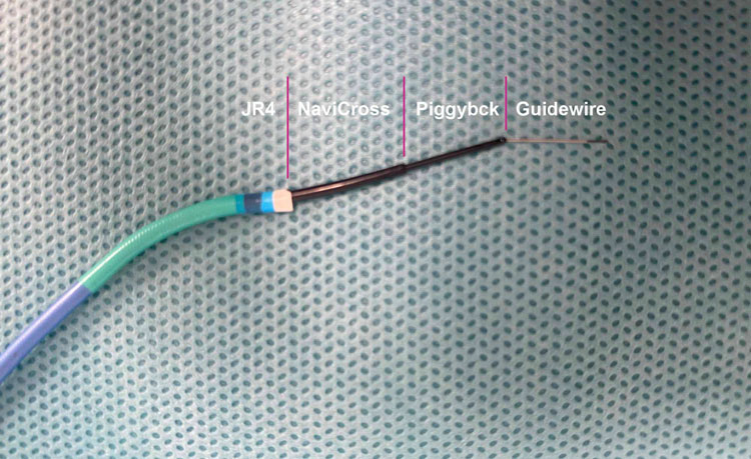
Puncture system. The puncture system was equipped outside to inside by a 6‐F 55‐cm JR4 guiding catheter, NaviCross microcatheter, Piggyback 0.035‐inch wire convertor, Astato XS 0.014‐inch coronary guidewire.

**Figure 3:**
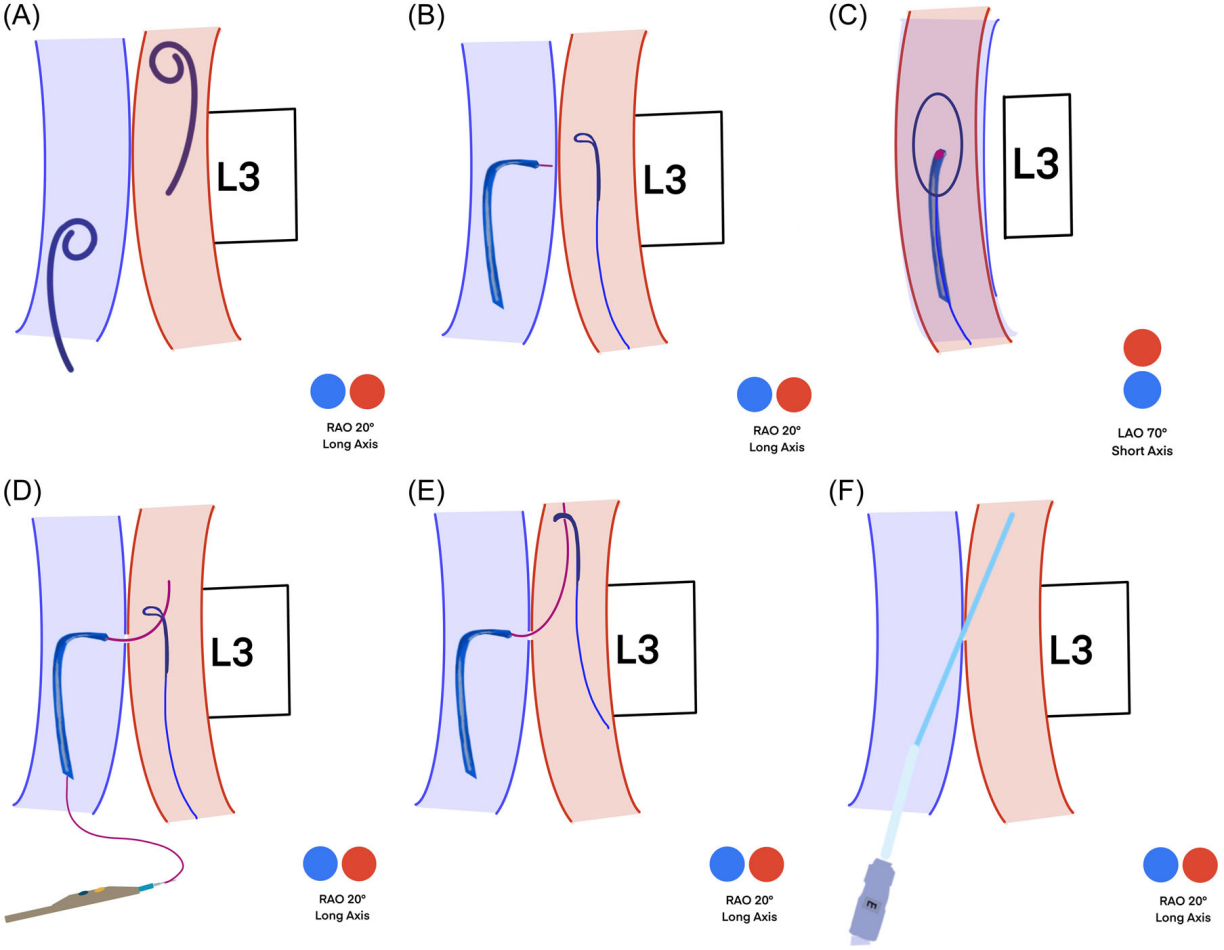
Schematic illustration of puncture technique. Simultaneous angiography is performed in long axis projection with two pigtails, which are higher in the artery and lower in the vein (A). The perpendicular puncture site is defined in both long (B) and short axes (C). Puncture is performed in the short axis (D). The snare system takes the guidewire toward the descending aorta (E). The balloon‐expandable sheath is introduced via stiff wire (F).

**Figure 4:**
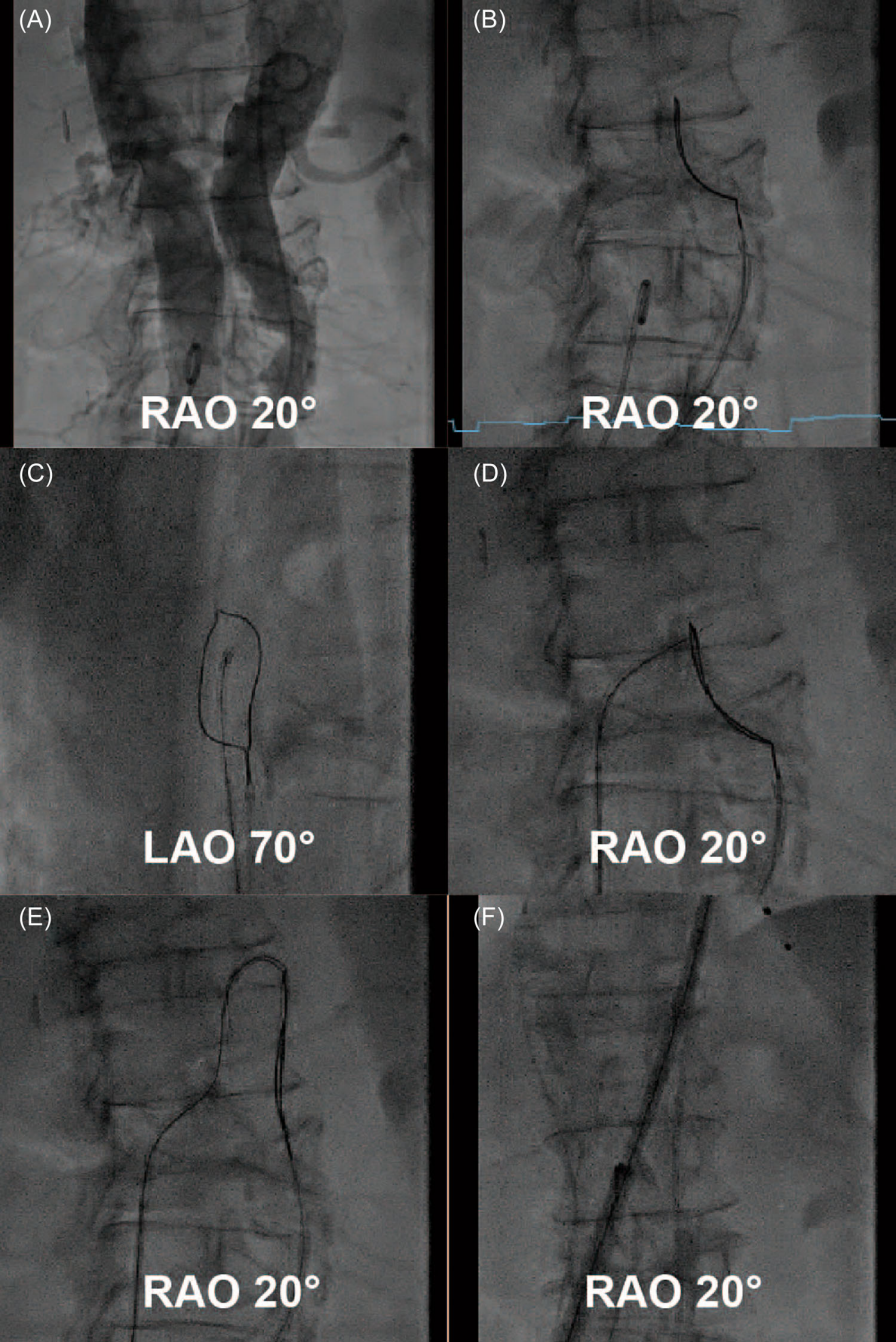
Puncture technique under fluoroscopy (A‐F)

The left femoral vein was introduced with a 6‐F sheath, pre‐closure with ProStyle device (Abbott Vascular), and an 8‐F sheath was introduced in the right femoral vein. The right femoral artery was introduced with a long 6‐F sheath. Simultaneous angiography was performed in RAO 20° projection (working view) with two pigtails, which were higher than L3 in the artery and lower than L3 in the vein. The upper part of L3 was defined as the puncture site. A goose‐neck snare system was exchanged against the pigtail and introduced from the femoral artery with overlap direction in RAO 20° projection and en‐face view in LAO 70°. A crossing system was equipped with a 6‐F 55‐cm JR4 guiding catheter (Cordis), NaviCross microcatheter (Terumo), Piggyback 0.035‐inch wire convertor, Astato XS 20 0.014‐inch coronary wire (Asahi Intec) from outside to inside and the distal end of the guidewire was de‐isolated and connected with an electrosurgery pencil (Central Illustration 1, Figure 2, and Table 1). After checking the guiding catheter is perpendicular to the short axis of LAO 70°, a short burn (approximal 1 s) with the electrosurgery pencil was performed to puncture the aortic wall in the long axis of RAO 20°. The coronary wire went through the cava‐aorta access, was snared, and pushed up toward the descending part of the thoracic aorta followed by Piggyback and NaviCross catheter. A backup Meier wire (Boston Scientific) was exchanged through the NaviCross, supporting the introduction of the eSheath (Edwards Lifesciences) straight through the cava‐aorta access (Figures 3 and 4).

**Table 1:** Material list for transcaval transcatheter aortic valve implantation

Category	Device
Crossing equipment	Gooseneck Snare, ev3
	Cordis RDC, RDC‐1, IM, JR4 guiding catheter, renal‐length (55 cm), 6, 7, or 8 Fr
	NaviCross microcatheter, straight tip, 90 cm length, 0.035″ ID
Closure	Piggyback wire convertor, 145 cm, Vascular Solutions
	Astato XS 20, Asahi, 300 cm
	Electrosurgical pencil
	Amplatzer Duct Occluder 1st generation 10/8, 12/10, and 8/6
	Agilis NxT SML curve 8.5 F × 71 cm
	0.014″ × 300 cm medium guidewire
Bailout	Medronic Reliant balloon—46 mm
	Advanta V12 (16 × 61 mm) covered stents

#### 2.3.2 Sapien 3 device deployment

As we described before, a 23‐mm balloon sizing technique was performed to choose the optimal valve size. Given that angiography showed a gap between hinge points of the annulus and the balloon, a 26‐mm Sapien 3 with 2‐ml underfilling was deployed.^2^

#### 2.3.3 Aorto‐caval access occlusion

**Figure 5:**
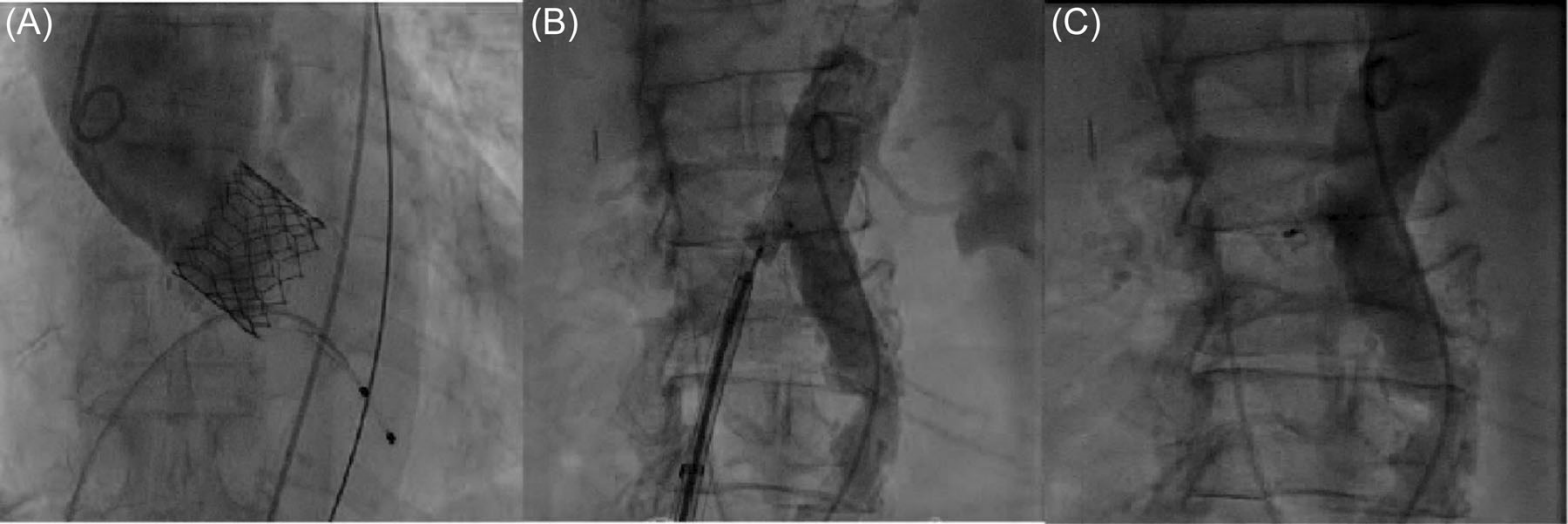
Shunt occlusion. After aortic root angiography showed the functional result of Sapien 3 (A), an Amplatzer Duct Occluder was deployed to occlude the aorto‐caval access (B). Final angiography showed complete occlusion (C).

A kink‐resistant 0.014 buddy wire as a safety backup to re‐access the aorta and an Agilias steerable sheath (Abbott Vascular) were advanced through the eSheath. A 10/8 mm Amplatzer Duct Occluder (ADO1‐1, St. Jude Medical) was introduced through the Agilias sheath and stepwise deployed coaxially during flection and then pulled back together with the eSheath. Angiography directly after placement showed mild residual shunt flow to IVC without signs of retroperitoneal bleeding. After 5 min and the application of protamine, complete closure of the puncture site was finally achieved (Figure 5). The procedural duration was 75 min.

## 3 DISCUSSION

Previous studies have shown the feasibility and safety of transcaval TAVI.^3^, ^4^, ^5^ Additionally, transcaval TAVI requires a similar setting in the operation room as transfemoral TAVI with good radiation protection and without the change of operators’ positions. In this case report, we describe the step‐by‐step procedural guidance. Based on our previous experiences,^3^, ^6^ we concluded that a good anatomical selection, precise cava‐aorta puncture, and complete shunt occlusion contribute to the success of the transcaval procedure.

### 3.1 Anatomy selection

As we described above, a good puncture site is needed for a cava‐aorta with a remarkable vertebral body of the spine as a reference under fluoroscopy. In particular, L3 could be divided into upper, middle, and lower parts to simplify the precise puncture site. Additionally, we recommended performing simultaneous angiography in both aorta and IVC under the long axis projection to check the predicted puncture site.

### 3.2 Cava‐aorta puncture

**Figure 6:**
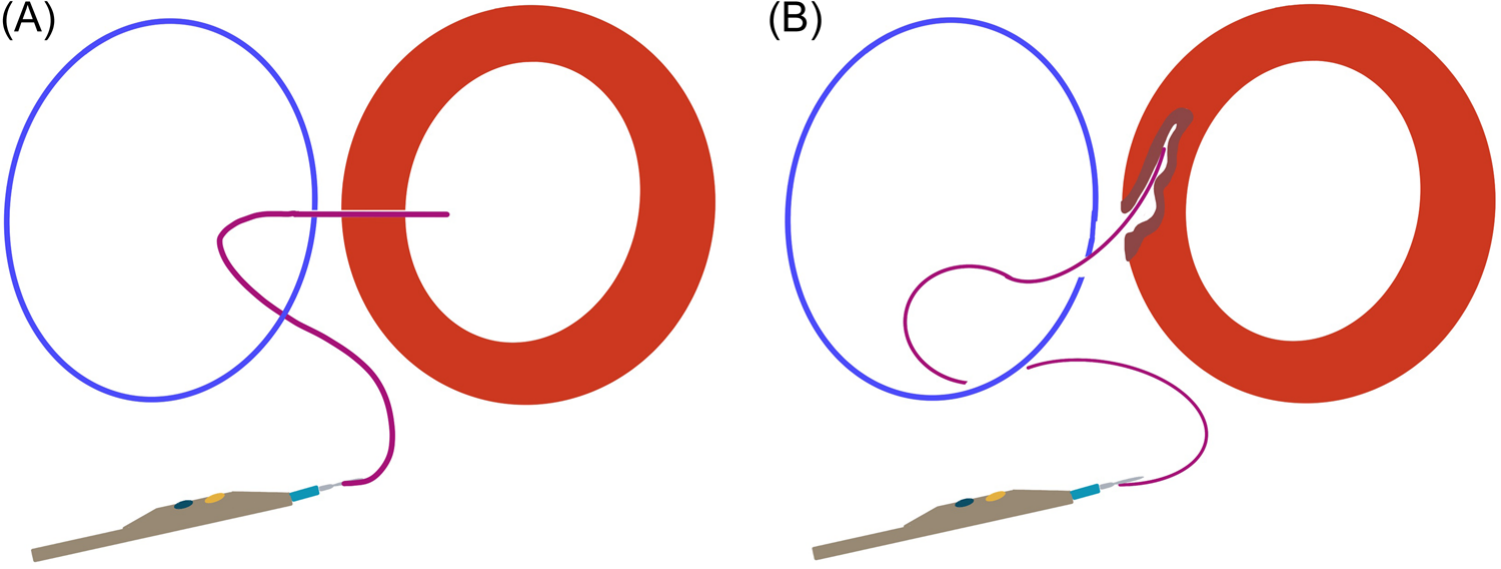
Perpendicular puncture. A perpendicular puncture ensures safety (A). An oblique puncture can cause aortic injury (B).

A perpendicular cava‐aorta puncture is a key to avoiding aortic injury (Figure 6). Therefore, we use predicted projections of cava‐aorta access on long and short axes. Projection in the long axis, depending on the anatomical situation around RAO 0°−30°, shows parallel IVC (left) and aorta (right) side by side, which is used as a working projection to show the immediate going through of the puncture wire. In this projection, we also perform simultaneous angiography to understand the anatomical situation and to place the goose‐neck snare in a perpendicular position. Projection on the short axis, mainly in RAO 60°−90° (90°—long‐axis angle), shows overlapped aorta and IVC. In this projection, we point our puncture system in the middle of the en‐face snare circle to ensure a perpendicular puncture (Central Illustration 1 and Figures 3 and 4).

**Central Illustration 1:**
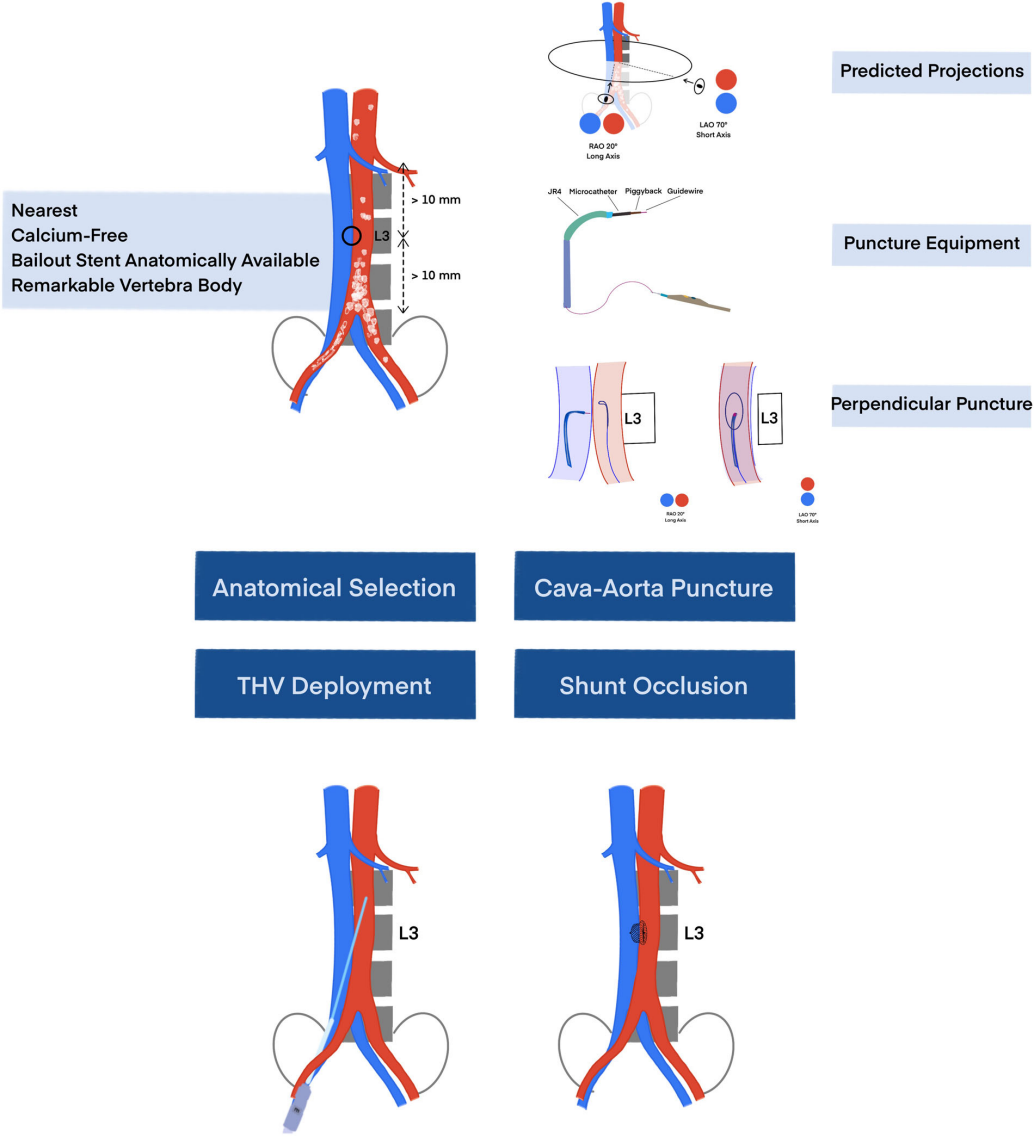
Transcaval TAVI. The ideal anatomic selection, perpendicular puncture with predicted projection and four‐component equipment, and complete shunt occlusion ensure the successful TAVI in the transcaval approach. THV, transcatheter heart valve.

A well‐equipped four‐component crossing system ensures the puncture of the aortic wall without injury to adherent tissues (Central Illustration 1). To avoid opposite aortic wall injury, it is important to stop burning direct as the guidewire crosses into the aorta. Also, the application of electro‐energy has to be stopped immediately if the wire crosses not straight through the wall and causes a cut instead of a puncture. Limited electro‐energy starting with 40 W is recommended.

### 3.3 Shunt occlusion

In our routine, we use an Amplatzer Duct Occluder; either 10/8 mm or 8/6 mm could be used depending on the used sheath size (14−20‐F). Muscular ventricular septal defect (VSD) Occluder could be used as an alternative.

To avoid an acute over preload of the right ventricle, a 25‐mm occlusion Reliant balloon (Medtronic World Medical) should be on standby to occlude the aorta in case of a retroperitoneal bleeding or hemodynamic relevant shunt. Also, an Advanta V12 (16 × 61 mm) covered stents (Getinge Maquet), which could be implanted over a small‐sized sheath (11‐F) was used as a backup in case of a failed occlusion of the cava‐aortic access.

## 4 FOLLOW‐UP

Postprocedural TTE showed well‐functional Sapien THV with a 4 mmHg gradient without a paravalvular leak.

## 5 CONCLUSIONS

Ideal anatomical selection, perpendicular cava‐aorta puncture, and complete shunt occlusion contribute to the successful TAVI in the transcaval approach. A further advantage of this alternative access is the same interventional setting as the transfemoral arterial TAVI.

## CONFLICT OF INTEREST

A.M.K. is a consultant and proctor for Edwards Lifesciences. The remaining authors have nothing to disclose.

## Supporting information

Video Title: Transcaval TAVI.
